# Enriched taxa were found among the gut microbiota of centenarians in East China

**DOI:** 10.1371/journal.pone.0222763

**Published:** 2019-10-22

**Authors:** Na Wang, Rui Li, Haijiang Lin, Chaowei Fu, Xuecai Wang, Yiming Zhang, Meifang Su, Peixin Huang, Junhua Qian, Feng Jiang, Hexing Wang, Lufang Jiang, Xin Yu, Jianxiang Liu, Yue Chen, Qingwu Jiang

**Affiliations:** 1 Key Laboratory of Public Health Safety of Ministry of Education, School of Public Health, Fudan University, Shanghai, China; 2 Taizhou Center for Disease Control and Prevention, Taizhou City, Jiangsu Province, China; 3 Deqing Center for Disease Control and Prevention, Deqing City, Zhejiang Province, China; 4 Yuhuan Center for Disease Control and Prevention, Wenling City, Zhejiang Province, China; 5 School of Epidemiology and Public Health, Faculty of Medicine, University of Ottawa, Ottawa, Ontario, Canada; Wageningen Universiteit, NETHERLANDS

## Abstract

**Background:**

Gut microbiota is closely related to age. Studies from Europe and the U.S. identified featured microbiota in different age groups for the elderly. Asian studies mainly focused on people living in longevity areas. Featured microbiota for the elderly people of different age groups, especially in the centenarian in the general population, has not been well investigated in China.

**Method:**

We conducted a comparative study by including 198 subjects of three age groups (65–70, 90–99, and 100+ years) in East China. Information regarding age, sex, height, weight, waist circumference, hip circumference, food preference, smoking status and alcohol consumption were collected by using a structured questionnaire. Fecal samples for each participant were collected as well. 16S rRNA gene sequencing were employed to analyze the gut microbiota composition. Logistic regression with LASSO feature selection was used to identify featured taxa in different age groups and to assess their potential interactions with other factors such as lifestyle.

**Result:**

The gut microbiota of the 90–99 year and 100+ year age groups showed more diversity, robustness, and richness compared with the 65–70 year age group. PCoA analysis showed a clear separation between the 65–70 and 100+ year age groups. At the species level, *Bacteroides fragilis*, *Parabacteroides merdae*, *Ruminococcus gnavus*, *Coprococcus* and *Clostridium perfringens* increased, but *Bacteroides vulgatus*, *Ruminococcus sp*.*5139BFAA* and *Clostridium sp*.*AT5* decreased in the 90–99 year age group. The age differences in gut microbiota were similar across the strata of smoking, alcohol consumption status and food preference.

**Conclusion:**

Our study demonstrated age differences in many aspects of gut microbiota, such as overall diversity, microbiota structure, and relative abundance of key taxa. Moreover, the gut microbiota of centenarian was significantly different from those of younger age groups of the elderly.

## Introduction

Gut microbiome has been proposed as a possible determinant of ageing because of its impact on metabolism and immunology[1–3]. Numbers of microorganisms inhabit the human intestine[4], resulting in improved nutrient absorption[5], accelerated physiological chemical transformation, enhanced host self-repair, and regulated immunity[4]. The preservation of host-microbes homeostasis can counteract inflamm-ageing[6], increase intestinal permeability[7], and decline in bone and cognitive health[8,9].

Human gut microbiota varies throughout a person’s lifespan and plays an important role in host health[10,11]. Recent studies on gut microbiota have revealed its associations with age, region[12,13], health condition[14], and antibiotic use[15]. Researchers have explored gut microbiota structure related to age by comparing their abundance based on culture- or PCR-based techniques. Studies on the differences in gut microbiota between the elderly and younger adults have yielded contradictory results, especially in the abundance of bifidobacterium, *Bacteroides*, and *Ruminococcus*[16–19]. However, most of the results above were based on the differences between adults and the elderly generally, while the gut microbiota variation of the elderly at different age has not been well studied.

A few studies have examined the effects of lifestyle and dietary factors on the association between ageing and gut microbiota[11,20,21]. Studies conducted among the elderly in Japanese and Europeans have observed siginificant changes in gut microbiota[11,22]. Most of the studies focused on people living in longevity regions (also called “Blue Zone”, where people could commonly live for 100 and more years) instead of general population[23,24], and evidences from East China were even less, though. Eastern china, as the most developed area, is facing a heavier burden of population ageing with its life expectancy being close to that of developed countries. Whearas, few studies focused on healthy ageing and microbiota. Cateloguing the specific features of microbiome that support healthy ageing is an essential step to identifying the microbial configurations that are implicated in longevity among eastern Chinese people. The cross-sectional study design as well as the limited sample size might be another concern for the results above. Moreover, most of the previous studies used culture- or PCR-based methods that only detected specific species of gut microbiota, and therefore could not reveal the whole picture of the microbiota structure[12,13,25].

In our study, we performed a population based case control study in East China to investigate the differences in gut microbiota among centenarians, the longevity, and the younger elderly, and to explore the association of ageing and gut microbiota. The 16S rRNA gene sequencing techniques were adopted to reveal more species as compared with traditional methods. We also recruited the subjects from general regions rather than the “Blue Zone” to better illustrate the association between longevity and microbiota. The purpose of the study was to help characterize specific microbiota signatures associated with age and lifestyle among the elderly and find the enriched taxa distributed in the longevity in China.

## Materials and methods

### Study population and sample collection

We enrolled 198 elderly subjects of three age groups (65–70, 90–99, 100+ years) from several communities in Deqing county (Zhejiang Province), Yuhuan county, and Haimen city(Jiangsu Province), East China. All the participants in this study were from our cohort built in 2015, and all of them were living in rural areas independently or with their children. We collected the fecal sample and finished questionnaire from August to September, 2017. All the subjects are generally healthy without medication. Members cannot meet our inclusion criteria will excluded from this study. The inclusion criteria is: (1)Han race, (2)No history of bowel-related surgery or congenital malformation. (3) No chronic or acute intestinal diseases, (4) No treatments with antibiotic and probiotic in previous 6 months. (5) The subjects have no relation with each other. (6) No adjustment with diet in recent 6 months. (7) No other chonic disease, such as diabetes, fatty liver, cirrhosis, kidney disease, and malignant tumor etc. Complete list of chronic disease we excluded could be found in supplemental materials and the related information were retrieved by self-reporting. The information of other chronic diseases we didn’t mention above or in supplemental materials were ignored as the incidence rates were relative low among our study population.

Those aged 90 years or above were defined as longevities, and 92 subjects (90–99 years: 52; 100+ years: 40) were enrolled. 106 residents aged 65–70 years in those three counties were selected and frequency-matched by sex and residential area with those aged 90 or older. The study protocol was approved by the Ethical Committee of Fudan University (Shanghai, China). All participants signed informed consent prior to sample collection. A structured questionnaire(Supplement material 1) was used to collect information on age, sex, height, weight, waist circumference, hip circumference, food preference, smoking status, as well as alcohol consumption. Trained technicians collected fecal samples for each participant in the morning with feces collection equipment. We collected at least 50 g of fecal sample each individual and frozen the fecal sample quickly in liquid nitrogen after defecation. Samples were placed in a mobile refrigeration device and transported back to the laboratory within 4 hours. All the samples were stored at -80°C and analyses were performed within one month.

### Sequencing and bioinformatic analysis

Total genomic DNA was extracted from fecal samples using a Powersoil DNA Extraction Kit (MoBio, Carlsbad, CA, USA) in 96-well format, and the 16S rRNA gene was amplified with barcoded fusion primers targeting the V3, V4, and V5 regions. We used Qubit 2.0 Fluorometer(Invitrogen, Carlsbad, CA) to test the concentration of the DNA. Library was contructed with kits of MetaVx (GENEWIZ, Inc., South Plainfield, Nj, USA). Amplicon pools were sequenced on a 2×150 bp Illumina MiSeq platform. Paired-end reads assembling were conducted with standard protocol. We also filtered low-quality reads with low sequencing score. After pairing the above-mentioned sequences, high-quality sequences were classified into multiple operational taxonomic units (OTUs) according to sequence similarity (> 97%).

The statistical differences in demographic and clinical characteristics were tested by using Pearson test and student *t*-test. BMI was divided into three subgroups (lower than 25, between 25 and 30 (exclusive), higher than 30 (inclusive)) instead of continuous variables. One-way analysis of variance (one-way ANOVA) and multiple t-tests with Bonferroni correction for continuous variables were used to test the differences in alpha diversity between age groups. A p-value less than 0.05 was considered statistically significant.

Reads were assembled using PANDAseq (v. 2.7)[26]. Trimmomatic (v. 0.30) was used to filter primers and adapter sequences[27]. USEARCH (v. 8.0) was employed to pair assembled and filtered reads[28]. The QIIME pipeline with RDP classifier Bayesian algorithm was used for taxonomic assignment with the SILVA_119 16S rRNA database. OTU classification, UniFrac analysis, and calculation of diversity metrics were also conducted with QIIME pipeline. Unweighted UniFrac distances were employed to assess the phylogenetic similarity of bacterial community pairs, taking into account OTU relative abundance or presence/absence, respectively. To visualize clustering of subjects based on pairwise distances, principal coordinate analysis (PCoA) plots were generated using the principal coordinates and labelled according to age groups. STAMP was employed to detect the difference of relative abundance at each level between age groups.

We also broadly compared bacterial taxa (phylum, family, species levels) between age groups. We limited our analysis of bacterial phyla to those with mean relative abundance ≥0.01%. For lower level taxa (family, genus and species), we limited our analysis to those with mean relative abundance ≥0.0001%. When comparing the non-normal distributed metrics between groups, the non-parameteric statistical method was employed. To explore the association of taxa relative abundance at species level with longevity, we conducted L1 penalized least absolute shrinkage and selection operator (LASSO) logistic regression implemented in R ‘glmnet’ package. This method built a parsimonious model which would only select the taxa having the strongest associations with the outcomeand this model could deal with the problem of collinearity[29]. We controlled covariates such as age, sex, BMI group, smoking status, alcohol consumption status, food preference in taxa selection process. Traditional logistic regression models were employed to evaluate their potential associations.

All statistical analyses were performed using R 3.5.1 software.

## Results and discussion

### Characteristics of participants

Due to the quality of collected samples, only 187 (90+ years: 92; 65–70 years: 95) samples were included in the analysis. Demographic characteristics of all participants are shown in Table 1. 61 longevities (66.3%) and 59 younger elderly (62.1%) were female. The average age of the longevities and younger elderly were 98.80 and 67.56 years, respectively. As compared with the younger elderly group, the longevity group were more likely to be obese (31.5% vs 3.1%). Waist-to-hip ratio, smoking, alcohol consumption and food preference were comparable between those groups. As compared with the younger age groups, centenarians (100+ years of age) were more likely to be nonsmokers and nondrinkers and to prefer vegetable or balanced diet rather than meat.

**Table 1:** Distribution of demographic characteristics according to age.

	Longevity(n = 92)					Younger Elderly^#^(n = 95)		
	90–99 yr (n = 52)		100+ yr (n = 40)		p value^¶^	No	Percent/Mean(SD)	p value*
	No	Percent/Mean(SD)	No.	Percent/Mean(SD)				
**Age(years)**		95.23±3.45		104.34 ± 3.12	0.03		67.56 ± 1.65	0.003
**Sex**								
** Male**	18	34.6%	13	32.5%	1	36	37.9%	0.87
** Female**	34	65.4%	27	67.5%		59	62.1%	
**BMI (kg/m^2^)**								
** < 25.0**	26	50.0%	23	57.5%	0.25	64	67.4%	<0.001
** 25.0–29.9**	4	7.7%	6	15%		28	29.5%	
** ≥30.0**	22	42.3%	11	27.5%		3	3.1%	
**WHR**		0.94±0.77		0.91 ± 0.56	0.44		1.06 ± 0.13	0.33
**Smoking status**								
** Ever Smokers**	8	15.4%	18	45.0%	0.004	10	10.5%	0.20
** Never Smokers**	44	84.6%	22	55.0%		85	89.5%	
**Drinking status**								
** Ever Drinkers**	8	15.4%	15	37.5%	0.03	16	16.8%	0.36
** Never Drinkers**	44	84.6%	25	62.5%		79	83.2%	
**Food Preference**								
** Vegetable preference**	16	30.8%	13	32.5%	0.13	27	28.4%	0.47
** Meat preference**	1	1.9%	5	12.5%		3	3.2%	
** Balanced-diet**	35	67.3%	22	55.0%		65	68.4%	

Abbreviations: BMI, body mass index; WHR; waist-hip ratio.

* *p* Values were based on t-test or test (two-sided) of the longevity and the younger elderly group.

^¶^ *p* values were based on t-test or test (two-sided) of elderly and centenarian group

^#^ “Younger Elderly” include the subject between 65–70 yrs.

### Diversity and distribution of fecal microbiota composition

A total of 1023 OTUs were identified for 187 samples. The numbers of OTUs that could only be found in some specific groups were 33 for the 90–99 year age group, 42 for the 100+ year age group and 46 for the 65–70 year age group, respectively.

Significant differences were observed between the 65–70 and 90–99 year age groups in richness as well as alpha index for diversity (p-value < 0.001 for all comparison). The community richness, measured by using Ace and Chao1 index, were lower in the 65–70 year age group (237.43 ± 66.07, 244.23 ± 69.75), as compared with the 90–99 year age group (279.74 ± 68.07, 283.48 ± 72.33) and the 100+ year age group (288.34 ± 65.68, 292.37 ± 68.26). Shannon and Simpson index suggested similar disparity. However, these indexes above were comparable between the 90–99 and 100+ year age groups.

**Fig 1:**
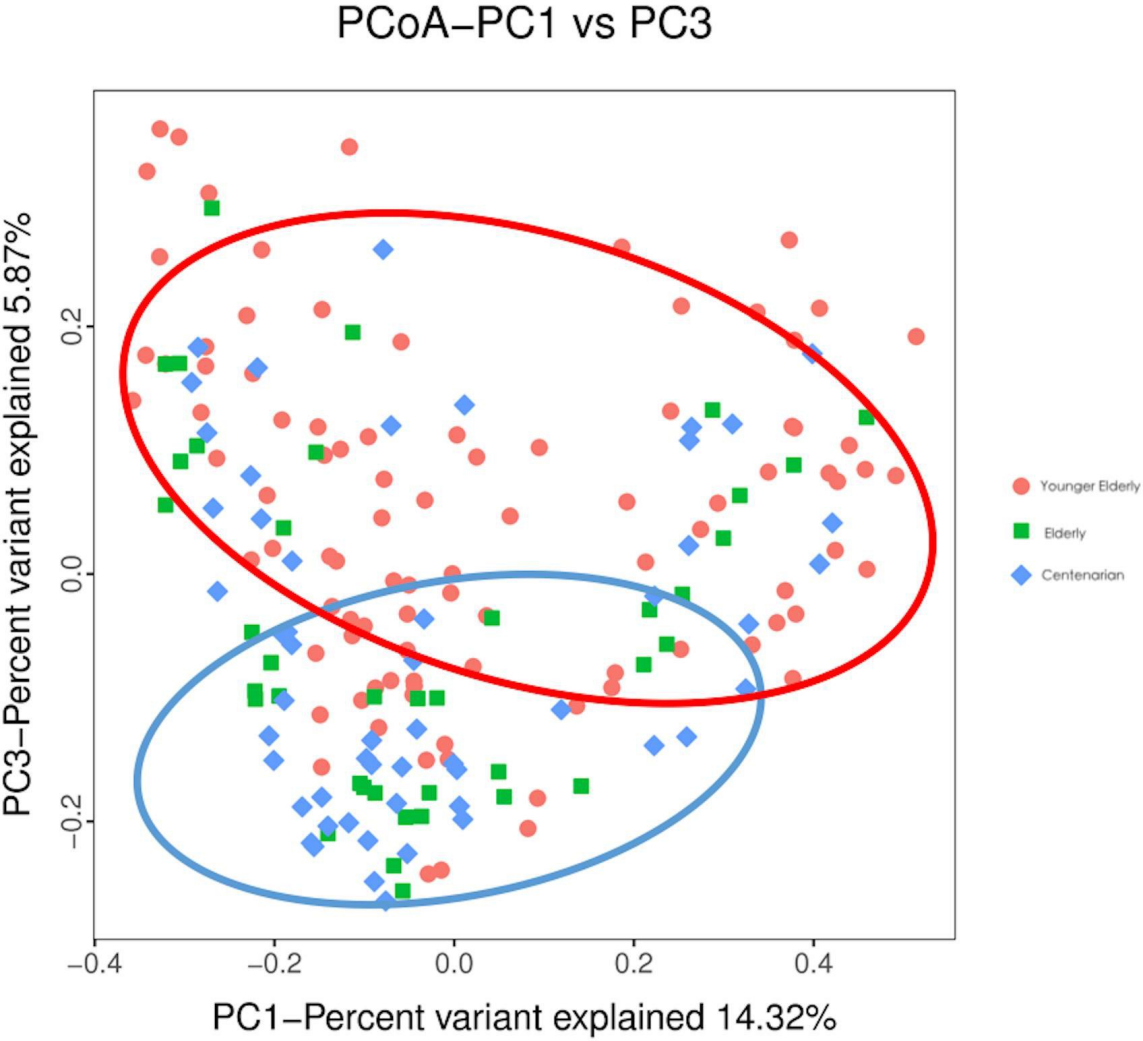
The first and third component of PCoA based on unweighted UniFrac distances*.

The first and third components of PCoA based on unweighted UniFrac distances were plotted to assess the similarity of microbiota distribution for these groups (Fig 1). After the PCoA conversion, a separation between the 65–70 and 100+ year age groups was observed, while the 90–99 and 100+ year age groups showed a similar distribution in the PCoA space. The percentages of variation represented by PC1, PC2 and PC3 were 14.32%, 6.76% and 5.87%, respectively.

### Fecal sample taxa composition

**Fig 2:**
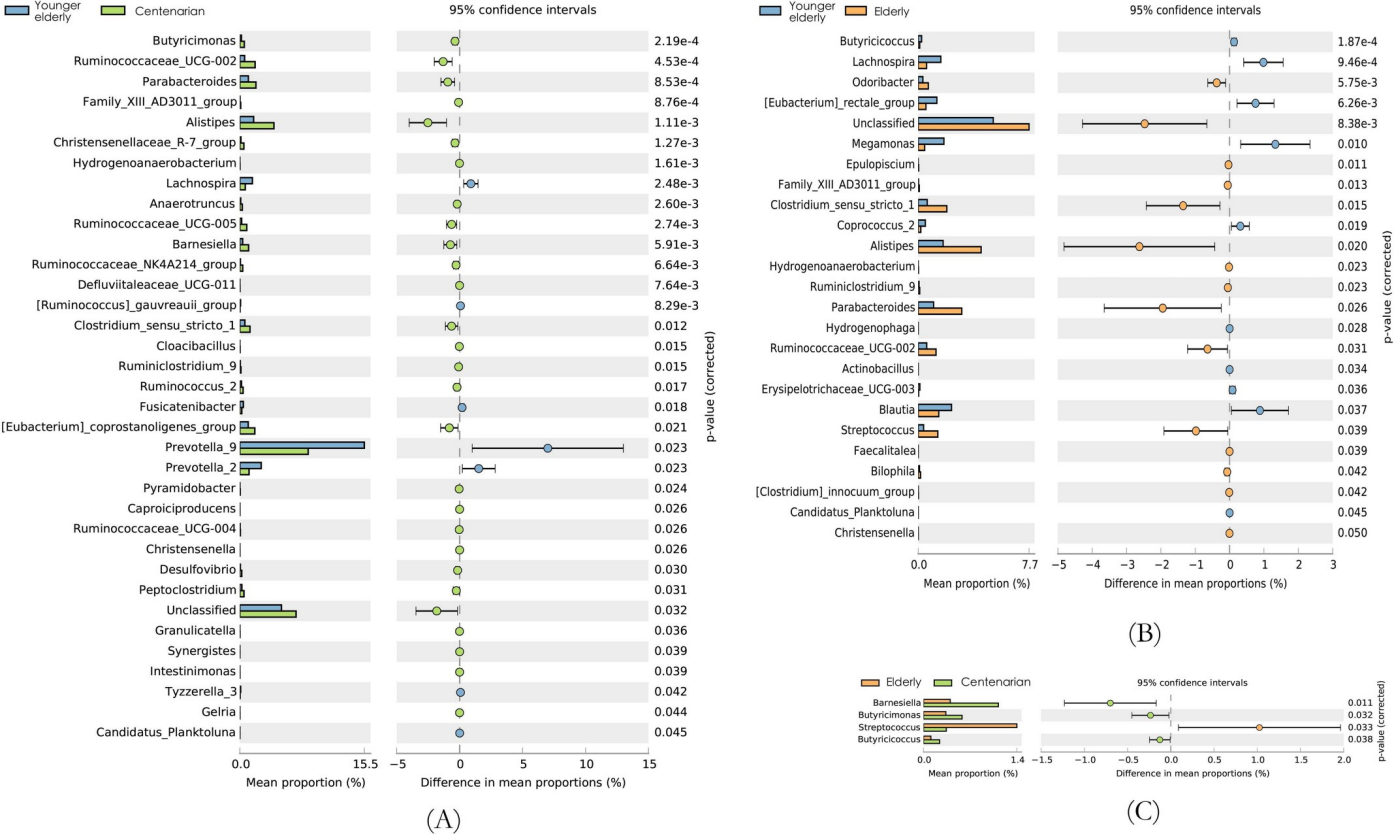
Relative abundance among three age groups at genus levels with p values. (A) Different relative abundance between the 65–70 and 100+ year age groups with 95% confidence interval and p values. (B) Different relative abundance between the 65–70 and 90–99 year age groups with 95% confidence interval and p values. (C) Different relative abundance between the 90–99 and 100+ year age groups with 95% confidence interval and p values.

Comparisons in the relative abundance of fecal bacterial taxa at the phylum, family (Table 2) and genus levels (Fig 2) between age groups were also conducted. At the phylum level, the dominant phylum taxa were *Bacteroidetes*, *Firmicutes* and *Proteobacteria* in each group. Most of the taxa showed no significant difference among the 3 age groups except for *Synergistetes* and *Verrucomicrobia*. The relative abundance of *Synergistetes* in the longevity group was 16 fold larger than that in the younger elderly group. At the family level, *Prevotellaceae*, *Lachnospiraceae* and *Porphyromonadaceae* were the taxa with higher relative abundance in the longevity group as compared with that in the younger elderly group (Table 2).

**Table 2:** The different relative abundance between the 65–70 and 90+ year age groups at phylum and family levels.

Taxa	Abundance in longevity	Abundance in younger elderly	Fold in change	p Value
**Phylum**				
***Synergistetes***	0.16(0.04–0.29)	0.01(0–0.03)	16	0.002
***Proteobacteria***	9.06(6.76–13.55)	7.69(5.34–8.49)	1.18	0.012
***Verrucomicrobia***	0.23(0–0.35)	0.15(0.11–0.21)	1.53	0.001
**Family**				
***Ruminococcaceae***	16.00(8.98–19.33)	14.20(13.22–16.83)	1.12	0.047
***Enterobacteriaceae***	6.72(5.45–7.88)	5.22(4.11–7.32)	1.29	0.003
***Rikenellaceae***	4.56(3.77–4.93)	1.78(0.97–2.14)	2.56	0.001

Compared with the 65–70 year age group, the 100+ year age group had a higher relative abundance of *Ruminococcaceae*, *Alistipes*, *Barnesiella*, et al. but lower relative abundance of *Lachnospira*, *Prevotella_9*, *Prevotella_2* (Fig 2). Although the differences were significant, the absolute abundance of these genus were not high (range from 3 to 150). For participants in the 90–99 year age group, higher relative and absolute abundance of *Clostridium_sensu_stricto_1*, *Parabacteroides* and *Streptococcus*, but lower abundance of *Megamonas*, *Blautia* and *Coprococcus_2* were observed as compared with the 65–70 year age group. As compared the 90–99 year age group, the 100+ year age group had higher abundance of *Barnesiella*, *Butyricimonas* and *Butyricicoccus* but the abundance of *Streptococcus*.

After adjustment for smoking, alcohol consumption, food preference and BMI group, *Bacteroides* and *Faecalibacterium* were associated with the longevity at the genus level by LASSO regression with L1 norm penalty (Table 3). At the species level, *Bacteroides fragilis*, *Parabacteroides merdae CL03T12C32*, *Ruminococcus gnavus*, *Coprococcus sp HPP0074* and *Clostridium perfringens* were positively correlated with longevity while *Bacteroides vulgatus*, *Ruminococcus sp 5139BFAA* and *Clostridium sp AT5* were negatively correlated with longevity (Table 3). Associations of these selected species with longevity did not differ by smoking, alcohol consumption status and food preference (all *p* value for interaction > 0.05).

**Table 3:** Relationship between bacterial taxa and longevity adjusted for smoking, alcohol consumption, body mass index (BMI) and food preference.

Factors	Crude OR	OR	95% CI	p-Value
***Bacteroides_fragilis***	9.87	5.98	1.63 to 25.59	0.01*
***Bacteroides_vulgatus***	0.25	0.16	0.04 to 0.65	0.01*
***Parabacteroides_merdae_CL03T12C32***	5.99	5.71	1.47 to 25.22	0.01*
***Ruminococcus***.***_gnavus***	2.34	7.95	1.59 to 48.14	0.02*
***Ruminococcus_sp***.***_5_1_39BFAA***	0.11	0.16	0.03 to 0.69	0.02*
***Clostridium_sp***.***_AT5***	0.14	0.23	0.06 to 0.82	0.03*
***Coprococcus_sp***.***_HPP0074***	7.57	6.44	1.24 to 38.98	0.03*
***Clostridium_perfringens***	5.84	4.08	1.12 to 16.48	0.04*
**Smoking status**	3.34	3.88	0.55 to 30.90	0.18
**Drinking status**	1.64	1.46	0.31 to 7.20	0.64
**BMI group**	0.73	0.87	0.45 to 3.12	0.76
**Food preference**	1.03	1.06	0.55 to 2.10	0.87

* p<0.05

In our study conducted in East China, gut microbiota variations with age were observed among the elderly. The 90+ year age group had significantly different characteristics in the overall abundance, diversity and composition of gut microbiota, as compared with the 65–70 year age group, suggesting the difference in gut microbiota might be a sign for ageing. We also found some enriched taxa between centenarians and younger ones, which may contribute to their longevity and structured microbiota with diversity.

Although the diet habit is not significantly different between case and control group, the longevity group have larger propotion of obesity subjects than the control group. Previous studies have shown that obesity causes many chronic diseases and reduces life expectancy, which is not inconsistent with our result. We have exclude the subjects with chronic disease out of our study, which may cause the decrease of level of harmful effects that obesity can bring about. Besides, the OR values of smoking status, alcohol comsuption, and food preference are not statistically significant after multi-factor adjustment. This can be explained by the same reason mentioned above. In further study, it is better to recruite the general population rather than the healthy subjects, which may lead to difficulties in investigating the interaction between lifestyle factors and microbiota structure and composition. Moreover, after multi-factor adjustment, the crude OR of some bacterials, such as *Bacteroides_fragilis* and *Ruminococcus*.*_gnavus* have been changed a lot, which means the interaction exists between bacterials and lifestyle factors and needs to be explored in further study.

Our findings partly support the note that age could have significant effects on the change of human gut microbiota throughout the whole life[1,10,11]. Ageing, the decrease of physical gastrointestinal function, unbalanced nutrition and the increase of frequent usage of antibiotic treatment could change gut microbiota significantly[1,10,18]. However, previous studies showed no notable changes in the composition of fecal microbiota for the elderly[30]. In our study, two *Bacteroides* strains, one *Clostridium* strain, and two *Ruminococcaceae* strains were significantly correlated with age among the elderly, and most of the identified microorganisms belonged to *Bacteroidetes* and *Firmicutes* phyla. Similar to other reports, *Proteobacteria*, *Fusobacteria*, and *Actinobacteria* phyla were less than 10% of the total community[31] and the community composition of gut microbiota at the phylum level was comparable to those previously reported for other populations[32].

Many studies have shown that the fluctuation and mean value of the alpha diversity of gut microbiota in human body is gradually decreasing with ageing[33]. As measured by richness alpha index and diversity alpha index, the alpha diversity index of subjects in the 90–99 and 100+ year age groups were significantly higher compared with the 65–70 year age group in our study. These results indicated the longevity group had more robust and abundant structure of gut microbiota, which was in line with the results from some other studies[34]. These differences could be partially represented by PCoA plot, in which a separation existed between the longevity group and the younger elderly group, suggesting that the younger elderly group had lower inter-individual variations of gut microbiota distribution. Study region and genetic background may explain the separation. The first and the second axes only explained less than one-fifth of the variances in both studies, indicating the effects of microbiota cannot be interpreted as several pivot factors and the bacteria with low relative abundance may contribute to the difference between the elderly and the longevity.

Researches on microbiota involving long-living individuals (centenarians) and using next-generation sequencing approaches, i.e. the golden standard of the modern microbiota analysis, are still very few. In 2015, Wang et al.[35]conducted a study on gut microbiota of people living in Bama County (Guangxi, China), one of the most notable Chinese “longevity hot spots”. The study showed several rearrangements in the gut microbiota of centenarians, such as a decrease in *Faecalibacterium* and *Akkermansia*, an increase in *Escherichia* and *Methanobrevibacter*, and a rearrangement in *Bacteroidetes*. However, only 24 samples were included in the study (8 centenarians and 16 controls), which may lead to inaccurate results. In our study, these taxa were not significantly different between the longevity group and the younger elderly group, except for *Bacteroidetes* rearrangement. As a “longevity hot spot”, natural environment and diet may be different, which can be another reason for the discrepancy.

The elderly living in Japanese or Russian longevous regions had relatively high numbers of bifidobacteria and *Lactobacillus*, and low amounts of clostridia[11,21]. The study in Bama County, also detected as high as 50% of bifidobacteria in total intestinal anaerobes[20]. bifidobacteria are regarded as a beneficial species in the colon[18], and high numbers of bifidobacteria have been observed in longevous people living in a specific region[11,20,21]. In our study, however, the abundance of bifidobacteria showed no substantial age-related variation, which was similar to the results from a study conducted by Zhao et al.[12]. Reasons for the inconsistency may include racial difference, genetic background and uncultured bacterial species. It has been estimated that approximately 75% of bacteria in the intestine are novel phylotypes[36]. The large fraction of novel bacteria in the intestine and their functions in host ageing should be paid more attention in future studies.

It has been proposed that the longevities had an increased number of *Bacteroides*[37,38], as well as a high level of *Bacteroides*-*Prevotella*[13]. *Bacteroides* is an essential bacterial genus in the colon because of its ability to digest polysaccharides and to utilize a wide variety of carbon sources[18]. These results suggested that these potential beneficial strains could be enriched and be “kept” from decreasing with age in the longevity's gut. We also detected one higher relative abundance of *Desulfovibrio* strain in the centenarian group. In the human gut, *Desulfovibrio* would reduce sulfate to produce hydrogen sulphide[39], which was potentially harmful to the host[11]. Whereas, the study performed in Italy by Biagi et al.[40] pointed out that *Desulfovibrio* could be opportunistic bacteria and enriched in elderly people. The actual function of this taxon and its interaction with the human body in different populations need to be further explored.

*Ruminococcaceae*, containing a large proportion of bacterial genera, can degrade dietary fiber, produce SCFA[41] as well as butyrate[19,42]. This bacterial genera may play an important role in the protection of the intestine[43]. The association of *Ruminococcus* with longevity remains contradictory[17,19]. The higher abundance of *Ruminococcus* in subjects on diet with rice preference had also been described by Salonen et al.[44], which was in line with our current study. The protection offered by *Ruminococcus* could partially explain the higher abundance in longevities. The enriched *Lachnospiraceae* in the younger elderly group in our study has also been observed in another study[45]. The health-promoting functions of *Lachnospiraceae* include participating in carbohydrate fermentation into short-chain fatty acids, CO_2_, and H_2_, resulting in increasing nutrients for the host and modulating colonic pH[46,47].

The strengths of our study include the selection of centenarians in a general population rather than in “longevity hot spots”, and relatively large sample size. The 16S rRNA gene sequencing techniques were used to reveal more species than traditional methods such as culture- or PCR-based methods. We also took into account for other factors (diet, smoking, drinking, and BMI subgroup) for the comparison of gut microbiota among age groups. By using L1 penalty, we focused on the taxa statistical relevant to longevity and quantified the likelihood of being the longevity of specific taxa by offering the OR values. Our study also has some limitations. As a comparative study, we could not determine whether the constitution of gut microbiota was changing all the time during the ageing process, or whether a specific structure could be maintained only by long-living subjects. Particular bacterial taxa are hypothesized to be involved in the establishment of new homeostasis with the ageing host, thus contributing to reach the extreme limits of human life[48].

## Conclusion

In conclusion, people with different age differed in many aspects of gut microbiota, such as overall diversity, microbiota structure, and relative abundance of key taxa. We also found the centenarians hold some key taxa that may contribute to their longevity, such as *Bacteroides fragilis*, *Parabacteroides merdae*, *Ruminococcus gnavus* and *Clostridium perfringens*. Some unclassified taxa may also contribute to longevity and need to be further explored.

## Supporting information

S1 Text Questionnaire about correlation between longevity and microbiota. (DOCX)

S2 Text The OTU representative sequence in our subject’s fecel sample. (FASTA)

S1 Table The OTU taxa table of representative sequence. (XLS)

S2 Table The meta information of the sequencing sample in our research including grouping information. (XLSX)
