# Supplementary material for: Enriched taxa were found among the gut microbiota of centenarians in East China
Source: PLoS One. 2019 Oct 22;14(10):e0222763. doi: 10.1371/journal.pone.0222763 (PMC6804974; doi:10.1371/journal.pone.0222763)
Supplement: S1 Text — (DOCX) [file pone.0222763.s001.docx]

**Questionnaire about correlation between longevity and microbiota（for those above 100 years old）**

1. Name：__________ ID：_____________ Sample Date-Time：___Month____Day_____Hour
2. Can you take care of yourself on your own？_____ 1.fully self-care 2. Partially self-care 3.cannot self-care
3. Birthday: year _____month Gender：_____ 1. male 2. female
   1. If you are female, you have _____ children？；you have abortion _____times？
4. Marriage status：_____1. Married 2. Widowhood 3. Divorced 4. Unmarried
5. Do you have following chronic disease？______________（multiply choice）

a. hypertension b. diabetes c. hyperlipidemia d. Chronic Gastritis e. enteritis f. Intestinal polyps

g. Cholecystitis, gallstones h. Fatty liver, cirrhosis i. Chronic kidney disease, kidney stones, kidney cysts

g. coronary disease k. Stroke l. Gout (high uric acid) m. Chronic bronchitis, asthma, COPD

n. malignancy（what type？） _________

1. Have you ever smoked for more than 6 months？____ 1. Yes 2. No（Jump to 7）
   1. If yes，How old are you starting to smoke？_____
   2. How many pack of cigarettes did you smoke？_____
   3. Do you smoke？_____ 1. Yes 2. No
      1. If no，How old are you quitting smoke？_____
2. Have you ever drink for more than 6 months？____ 1. Yes 2. No（Jump to 8）
   1. How old are you starting to drinking？_____
   2. Do you drink？____ 1. Yes 2. No
      1. If“Yes”，How many times do you drink ____ ?
3. What is your diet in recent 12 month? _______1. Mostly vegetable 2. Mostly meat 3. Balanced
4. Have you made major adjustments to your diet in recent years？____ 1. Yes 2. No
   1. If the answer is “Yes”，When did you start to change______；The reason is __________
5. When is your most recent medical examination：______Year ____Month
   1. Result：

Height_____；Weight_____Kg；waistline _____cm；hipline _____cm；blood pressure _____/_____mmHg；blood sugar______mmol/L

Signature:______________

1. **Questionnaire about correlation between longevity and microbiota（for those below 100 years old）**Name：__________ ID：_____________ Sample Data-Time：___Month____Day_____Hour
2. Can you take care of yourself on your own？_____ 1. Fully 2.Partially 3.Cannot Gender：_____ 1. Male 2. Female
   1. If the answer above is Female, you have _____ children？How many times you aborted _____？
3. Do you have the following chronic disease（diagnosed by doctor）？______________（Multipl Choice）

a. hypertension b. diabetes c. hyperlipidemia d. Chronic Gastritis e. enteritis f. Intestinal polyps

g. Cholecystitis, gallstones h. Fatty liver, cirrhosis i. Chronic kidney disease, kidney stones, kidney cysts

g. coronary disease k. Stroke l. Gout (high uric acid) m. Chronic bronchitis, asthma, COPD

n. malignancy（what type？） _________

1. What is your diet in recent 12 month? _______1. Mostly vegetable 2. Mostly meat 3. Balanced
2. Have you made major adjustments to your diet in recent years？____ 1. Yes 2. No
   1. If the answer is “Yes”，When did you start to change______；The reason is __________
3. When is your most recent medical examination：______Year ____Month
   1. Result：

Height_____；Weight _____Kg；waistline _____cm；hipline _____cm；blood pressure _____/_____mmHg；blood sugar ______mmol/L

Signature: _____________
